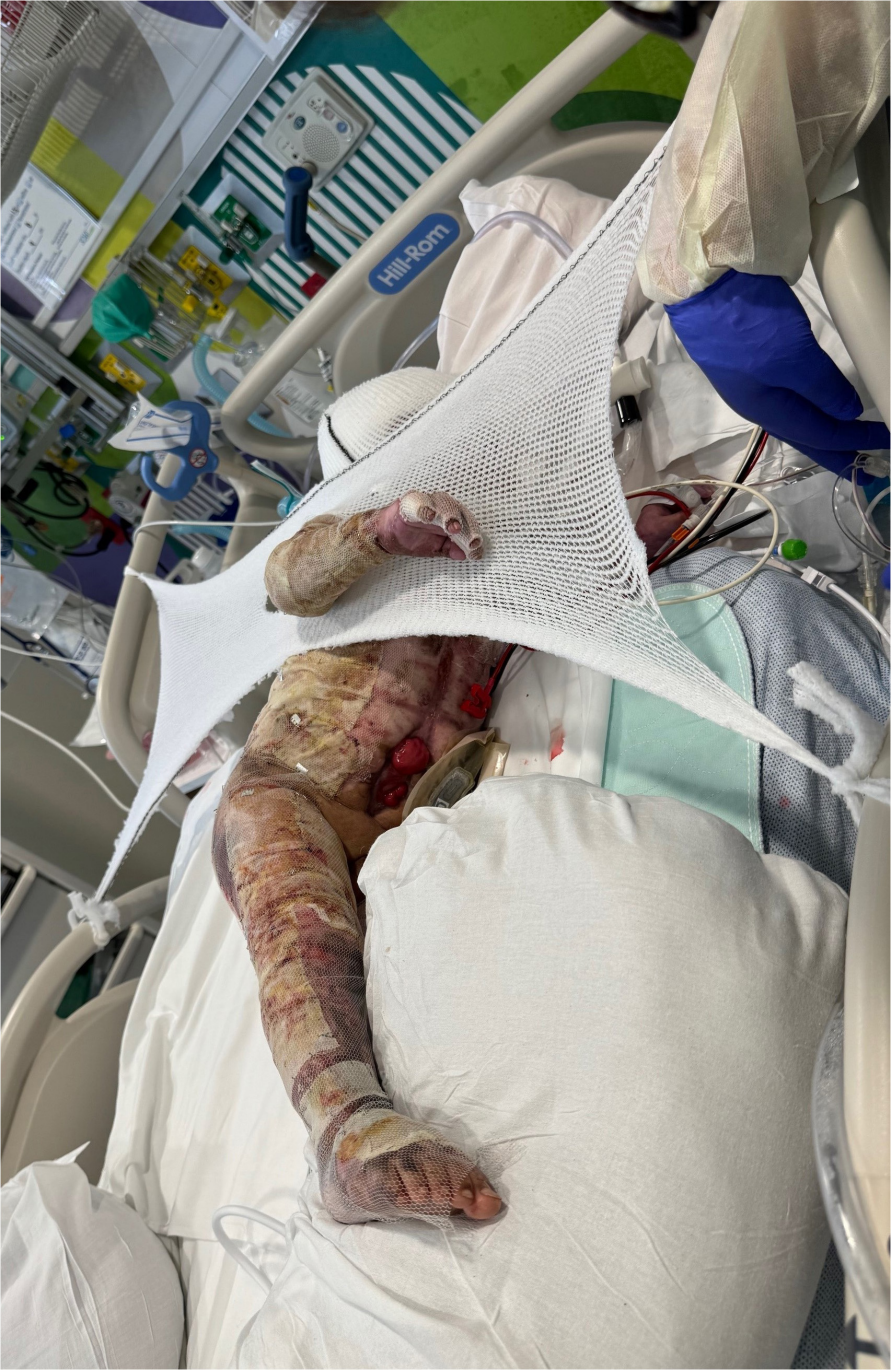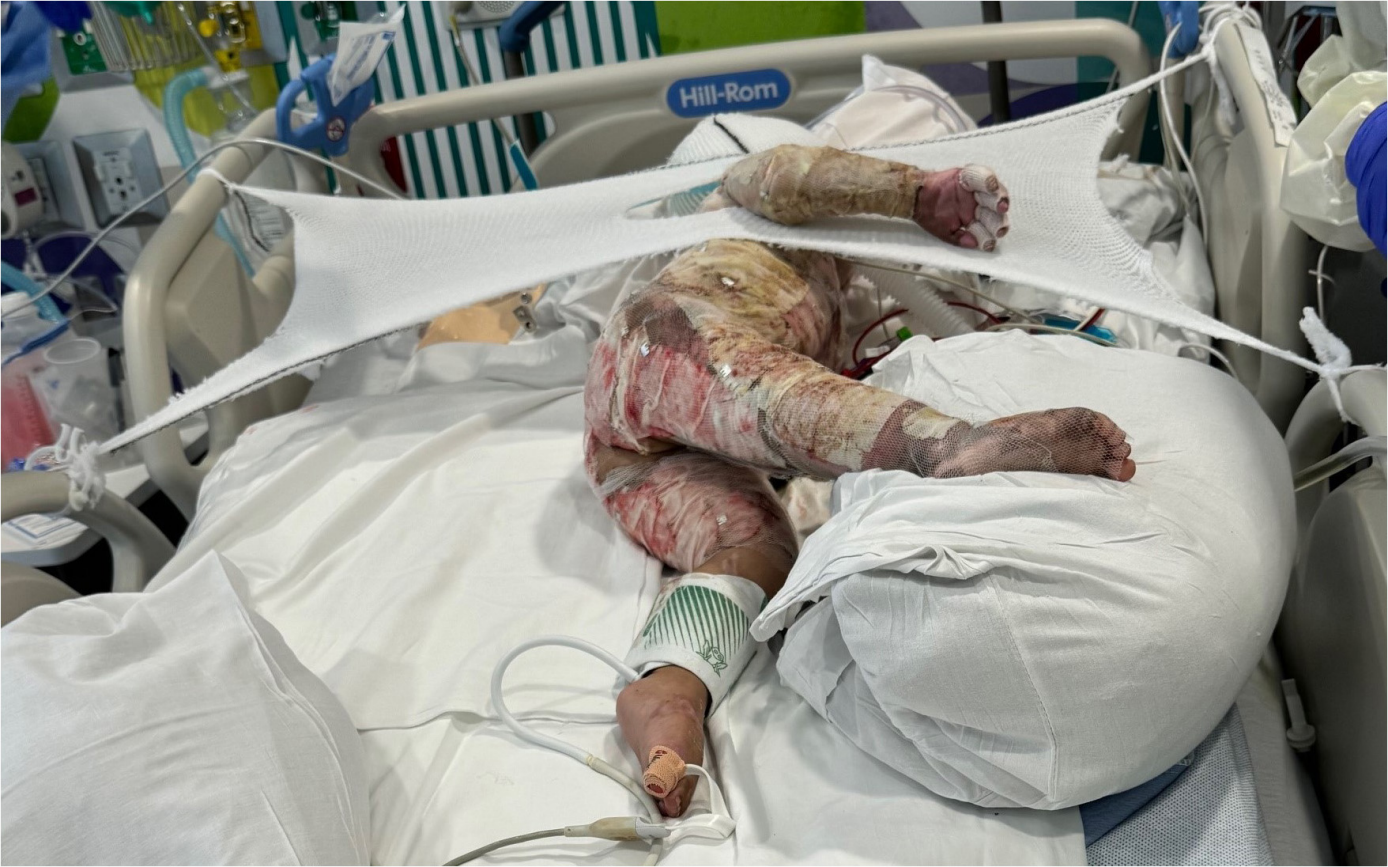# 899 Creativity in Treating Pediatric Burn Patients: An Essential Need

**DOI:** 10.1093/jbcr/iraf019.430

**Published:** 2025-04-01

**Authors:** Alice Fagin

**Affiliations:** Shriners Children’s - Ohio

## Abstract

**Introduction:**

Pediatric burn patients account for approximately 22.5% of all burns in the United States. Severe burns are an even smaller percentage. Medical equipment innovation and regulation is a time intensive and exhaustive process. Companies are most likely to produce devices for the largest populations of patients. This creates a difficulty in providing age and size appropriate devices for our severely burned pediatric population.

**Methods:**

Here we present a case of a 60% TBSA burned 21-month-old child who required multiple excisions with ultimate skin closure accomplished with widely meshed autograft, epithelial autograft, and cultured epidermal autograft. We discuss challenges related to his care and positioning and the creative methods and devices created to facilitate healing. A literature review reveals feasibility of innovation and limitations to large scale pediatric burn product production.

**Results:**

The innovative positioning supports required modification as patient’s positioning needs changed. Overall, the positioning worked well and the patient healed appropriately.

**Conclusions:**

Creativity and innovation are essential components of burn care overall, but even more so for pediatric patients. Positioning devices, in particular, can be problematic as they are mostly made for adult sized patients. While there is a need, the population is small enough and diverse enough that large scale product production is not feasible or reasonable. Innovation with a multidisciplinary approach will yield best results.

**Applicability of Research to Practice:**

Burn care is individualized and patients have diverse needs. The ability to innovate and re-purpose common devices is essential for effective burn care.

**Funding for the Study:**

N/A